# The Genotypic and Phenotypic Spectrum of *GOSR2* Mutations: Clinical and Pathophysiological Insights

**DOI:** 10.1002/jimd.70115

**Published:** 2025-11-20

**Authors:** Sjoukje S. Polet, Elisabeth Z. Siegal, Sabine A. Fuchs, Marina A. J. Tijssen, Tom J. de Koning

**Affiliations:** ^1^ Expertise Centre for Movement Disorders, Department of Neurology, University Medical Centre Groningen University of Groningen Groningen the Netherlands; ^2^ Department of Metabolic Diseases, Wilhelmina Children's Hospital University Medical Center Utrecht Utrecht the Netherlands; ^3^ Department of Genetics, University Medical Centre Groningen University of Groningen Groningen the Netherlands; ^4^ Pediatrics, Department of Clinical Sciences Lund University Lund Sweden

**Keywords:** *GOSR2*, hearing loss, muscular dystrophy, progressive myoclonus ataxia, progressive myoclonus epilepsy, *SNARE* proteins

## Abstract

North Sea‐Progressive Myoclonus Epilepsy (NS‐PME) is a progressive neurological disorder, initially only associated with the homozygous *GOSR2* founder mutation (c.430G>T; p.Gly144Trp). Clinical symptoms include untreatable early‐onset ataxia, cortical myoclonus and epilepsy. Recently, the spectrum of *GOSR2* mutations and associated phenotypic variability has expanded. To improve care and to facilitate genotype–phenotype predictions for NS‐PME patients, we systematically reviewed all reported *GOSR2* mutations, clinical phenotypes, and pathophysiological findings. A narrative review literature search was conducted in PubMed, EMBASE, and Web of Science (1985—August 2024) using the keywords “GOSR2”, “GS27 protein”, “Bos1”, and “Membrin”. Only studies in English and specifically studies on GOSR2 function, pathogenic variants, clinical manifestations, and potential therapies were included. A total of 42 patients with 11 different *GOSR2* mutations were identified. Three main phenotypes were observed: progressive myoclonus ataxia/epilepsy (PMA/PME), congenital muscular dystrophy, and hearing loss. Orthopedic abnormalities were frequently reported. Intercurrent infections or fever often led to a worsening of symptoms. Glycosylation defects were reported in several compound heterozygous *GOSR2* variants. Molecularly, *GOSR2* mutations result in (partial) loss of function of the GOSR2/SNARE complex, with mutation severity and the involvement of specific isoforms contributing to phenotypic variability. *GOSR2* mutations lead to progressive neurological disorders, primarily characterized by myoclonus ataxia/epilepsy, muscular dystrophy, and hearing loss. The genotypic background of NS‐PME has expanded with pathogenic biallelic GOSR2 variants beyond the original homozygous founder mutation. Understanding the clinical spectrum and molecular mechanisms of GOSR2‐related diseases may facilitate more targeted treatment strategies as well as better‐informed phenotype predictions.

## 1 Introduction

North Sea‐Progressive Myoclonus Epilepsy (NS‐PME) is a childhood‐onset neurological disorder caused by mutations in the *GOSR2* gene. In 2011, the first report on NS‐PME characterized a group of persons with the homozygous founder mutation c.430G>T, (p. Gly144Trp) mutation [1]. The phenotype of NS‐PME is homogenous, consisting of early‐onset ataxia, followed by the onset of progressive myoclonic jerks and epilepsy, with most persons with NS‐PME being wheelchair bound around their teenage years [2]. The ancestors of a large cohort with this pathogenic variant all originated from countries bordering the North Sea, indicating a founder effect [3]. Following the initial report of NS‐PME in 2011, subsequent studies have revealed other mutations in the *GOSR2* gene. Some of the pathogenic biallelic *GOSR2* variants are associated with the NS‐PME phenotype. However, an expanded neurological phenotype has been described as well, including severe muscular dystrophy, and involvement of other organ systems including the musculoskeletal, cardiovascular and auditory system, indicating multisystem involvement of the gene beyond brain tissue [4, 5].

The *GOSR2* gene (also known as *GS27/membrin* in *Drosophila melanogaster* /fruitfly and *bos1* in *Saccharomyces cerevisiae* /yeast), was first reported in 1997 and encodes a soluble N‐ethylmaleimide‐sensitive factor attachment protein receptor (SNARE) protein [6, 7]. SNARE proteins are a family of proteins critical to the process of membrane fusion, an essential cellular mechanism fundamental to numerous intra‐ and extracellular functions, including exocytosis, endocytosis, viral entry, and fertilization [8]. For example, neurotransmitter release at nerve terminals is mediated by the exocytosis of synaptic vesicles, which undergo docking and fusion with the presynaptic membrane prior to their release [9]. SNARE proteins are well conserved throughout various eukaryotic species [10]; in humans, 36 SNARE family members have been identified and studied [11].

To date, mutations in 13 of these SNARE encoding genes have been linked to clinical conditions involving multiple organ systems, especially the nervous, immune and endocrine system [12]. However, the phenotypic spectrum remains poorly defined and the pathophysiological mechanism underlying the clinical manifestations in diseases caused by mutations in SNARE protein encoding genes and in particular in *GOSR2* still remains largely elusive.

To address the latter, we need to fully comprehend the clinical entities associated with the expanding list of mutations in *GOSR2* and gain deeper understanding of the molecular consequences on GOSR2 functioning. Ultimately, this knowledge can help improve treatment strategies for GOSR2‐related conditions. Therefore, we provide a comprehensive overview of: (1) GOSR2‐associated conditions and their clinical features; (2) Current understanding of the function of the GOSR2 protein; (3) Molecular and cellular effects of mutations in *GOSR2* including genotype–phenotype relations; and (4) Pathophysiological insights derived from cellular and animal studies with altered GOSR2 function/concentrations.

## 2 Materials and Methods

PubMed, EMBASE, and Web of Science databases, including review studies and conference abstracts were searched, using at least one of the following keywords: “GOSR2”, “GS27 protein”, “Bos1” or “Membrin”. Additionally, some articles were identified through citation tracking. Only studies published in English between 1985 and August 2024 were included. Studies focusing on the general functioning of the GOSR2 protein/Membrin, previously reported mutations s in *GOSR2*, clinical manifestations associated with mutations in *GOSR2*, and therapies for GOSR2‐associated conditions were included. Studies addressing GOSR2 in the context of cellular studies unrelated to the direct function were excluded.

## 3 Results

### 3.1 *GOSR2* ‐Associated Conditions: Clinical Phenotypes

**FIGURE 1:**
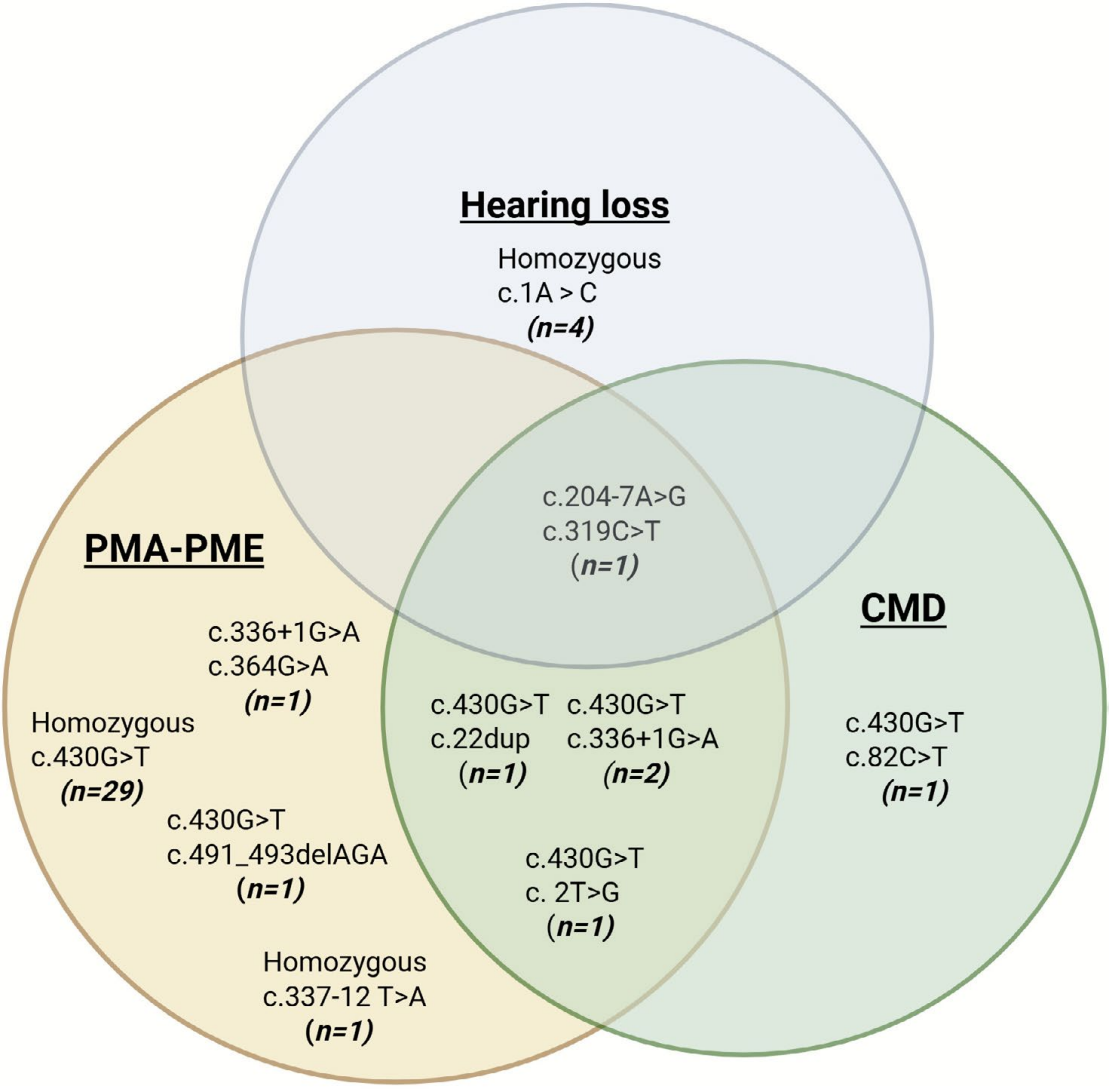
Overview of the three main phenotype entities associated with the reported *GOSR2* pathogenic variants. In brackets the number of patients reported with the variant is indicated. CMD = congenital muscular dystrophy; PMA = progressive myoclonus ataxia; PME = progressive myoclonus epilepsy. Created in BioRender by Polet S (https://BioRender.com/djc581t).

In total, we identified 42 reported persons with 11 different *GOSR2* variants (including missense, non‐sense and in‐frame deletions) and 15 different single nucleotide variants (SNV)/haplotypes. Clinical features primarily involved neurological symptoms, including movement disorders, epilepsy and congenital muscular dystrophy (CMD), or combinations hereof, but also other systems/tissues/organs were involved. Figure 1 displays the three main clinical phenotypes: (1) Progressive Myoclonus Ataxia, PMA‐Progressive Myoclonus Epilepsy, PME (isolated 32/42 + combined 4/42); 2. Congenital Muscular Dystrophy, CMD (isolated 1/42 + combined 4/42) and 3. Hearing loss (isolated 4/42 + combined 1/42). An overview of the reported mutations and their main associated clinical features is shown in Table 1.

**TABLE 1:** Overview of *GOSR2* pathogenic variants with the number of cases reported, phenotypic features and molecular and functional consequences of the pathogenic variants.

Genotype	No. cases	Molecular consequence	Functional consequence	Phenotype	References
Homozygous: c.430G>T (p. Gly144Trp)	29	Missense mutation	Partial loss of function of GOSR2 Interference with SNARE hydrophobic core	Classical phenotype PMA/PME *Other* Scoliosis, Developmental delay Areflexia Hypotonia Hypothyroidism	Corbett et al. [1] Boissé Lomax et al. [3] Van Egmond et al. [13] Anderson et al. [14] Völker et al. [15] Polet et al. [2] Dafsari et al. [16]
Compound heterozygous: *c.430G>T* c.336 + 1G>A (p.?)	2	Splice site mutation, Donor loss 0.99 according to Splice AI.	Unknown	Classical phenotype PMA/PME (*n = 1*) and Mixed phenotype: CMD and PMA‐PME (*n = 1*) *Other* Developmental delay Hypotonia Optic nerve atrophy	Tsai et al. [4] Kleinhenz et al. [17]
Compound heterozygous: *c.430G>T* c.491_493delAGA (p.Lys164del)	1	In‐frame deletion	Partial loss of function of GOSR2 Interference of hydrogen bonds between GOSR2 and syntaxin‐5	*Classical phenotype PMA/PME* *Other* Scoliosis Areflexia	Praschberger et al. [18]
Compound heterozygous: *c.430G>T* c.2T>G (p.Met1Arg)	1	Missense initiator codon variant. Likely resulting in the use of an alternate start codon with elimination of 18 amino acids from the amino‐terminus	Unknown	*Mixed phenotype: CMD and PMA‐PME*	Larson et al. [19]
Homozygous: c.337–12T>A (p.Asp113‐Lys159del)	1	Affecting splicing of exon 5 Resulting to in‐frame deletion of 47 amino acids in the SNARE domain of GOSR2	Unknown	*Classical phenotype PMA/PME* *Other* Severe microcephaly Hypotonia Hypertrichosis	Smorag et al. [20]
Compound heterozygous *c.430G>T* c.82C>T (p.Gln28Ter)	1	Non‐sense variant	Predicted to result in protein truncation or nonsense mediated decay	*Predominant CMD* *Other* Abnormal EEG without clinical seizures Hypotonia Developmental delay Subtle arthrogryposis	Henige et al. [21]
Compound heterozygous c.204‐7A>G (p.?) c.319C>T (p. Arg107Ter)	1	Suggested to create an alternative 3′ splice site à Splice AI shows 0.98 acceptor gain. In ClinVar denoted as likely benign. Nonsense variant Predicted to introduce a premature stop codon	cDNA analysis of muscle‐derived RNA showed that approx. 40% of the transcripts were abnormally spliced, allowing for residual normal splicing. Predicted to be a loss‐of‐ function variant	*Mixed classical phenotype, CMD and hearing loss* *Other* Insulin dependent diabetes Areflexia	Stemmerik et al. [22]
Compound heterozygous c.336 + 1G>A (p.?) c.364G>A, p.Glu122Lys	1	Splice site mutation Missense mutation	Predicted to distrupt the IxM motif for packaging of GOSR2 and syntaxin‐5 into COPII coated vesicles Suggested to have minimal impact on SNARE assembly	*Classical phenotype PMA/PME* *Other* Developmental delay	Dafsari et al. [16]
Compound heterozygous *c.430G>T* c.22dup (p.Thr8fs)	1	Frameshift mutation: Single‐ base pair duplication, predicted to introduce a frame shift starting at codon 8, resulting in a premature stop codon 54 codons downstream	Unknown	*Mixed phenotype: CMD and PMA/PME* *Other* Bilateral club feet Plagiocephaly Scoliosis Developmental delay	Arroyo et al. [23]
Homozygous c.1A>C (p.Met1Leu)	4	Missense initiator codon variant. Likely pathogenic.	Reduction of GOSR2 protein	*Hearing loss phenotype* *Other* Febrile seizures	Aburayyan et al. [5]

*Note:* After the first report, the c.430 G>T has been indicated in italic font, meaning that the functional/molecular consequence has been discussed, and the molecular/functional consequence information does not apply for this mutation.

Abbreviations: CMD, congenital muscular dystrophy; PMA, progressive myoclonus ataxia; PME, progressive myoclonus epilepsy.

### 3.2 Classical Phenotype: Progressive Myoclonus Ataxia/Progressive Myoclonus Epilepsy (PMA/PME)

The classic phenotype associated with mutations in *GOSR2* typically includes early‐onset ataxia, progressive myoclonic jerks and epilepsy. As such, this phenotype falls within the spectrum of Progressive Myoclonus Ataxia (PMA) and Progressive Myoclonus Epilepsy (PME). The most common mutation in *GOSR2* associated with this phenotype is the homozygous missense mutation c.430G>T (p.Gly144Trp) [3], and all persons with this mutation either in compound heterozygous or homozygous form display PMA‐PME. Three other persons with different genotypes also display the classical phenotype of PMA‐PME: homozygosity for c.337‐12T>A (p.Asp113‐Lys159del) (*n* = 1), compound heterozygosity for the known c.430G>T and a novel c.491_493delAGA (p.Lys164del) (*n* = 1), and compound heterozygosity for c.336 + 1G>A (p.?) and c.364G>A, (p.Glu122Lys) (*n* = 1) (Figure 1).

Ataxia usually presents at the age 2 of years, often triggered by intercurrent illness or fever, although the person with c.336 + 1G>A + c.364G>A mutations developed ataxia at age 12. Ataxia was absent in the homozygous c.337‐12T>A mutation. Ataxia is frequently accompanied by delayed motor development. Myoclonus, characterized by brief involuntary jerks, is the predominant feature of the classical phenotype and progressively worsens by becoming generalized or multifocal. Exacerbating factors include fever, emotional stress, and heat [24]. Episodes of motor deterioration are also triggered by infection and fever [2, 18]. Neurophysiological findings suggest cortical hyperexcitability, with characteristics such as giant somatosensory evoked potentials (SSEPs), photosensitivity, and cortico‐muscular coherence [25]. Drop attacks, likely due to negative myoclonus, have been observed as well [13]. Seizures typically begin before or during the second decade and include generalized motor seizures (tonic–clonic and myoclonic) and generalized non‐motor seizures (absences) [2, 3]. Seizure severity varies among persons with the classical phenotype ranges from rare occurrence of seizures to episodes with status epilepticus. EEG typically shows generalized spike‐and‐wave activity with posterior predominance and pronounced photosensitivity [2, 3]. Dystonia, characterized by abnormal muscle contractions leading to repetitive movements or postures, has been observed in a small number of persons with NS‐PME (*n* = 2). Despite significant motor involvement, cognitive function remains largely preserved. Mild cognitive decline or intellectual disability is reported in some [2, 3, 16, 18, 20].

Other features associated with the classical phenotype are skeletal deformities including scoliosis, pes cavus, syndactyly and clubfoot. Scoliosis is the most common deformity and some persons require surgical intervention to prevent complications of severe scoliosis. Hypotonia, mildly elevated Creatine Kinase (CK) levels (range 61–2900 U/L) and hyporeflexia or areflexia are consistently observed. However, muscle strength remains normal, muscle biopsy histology is unremarkable, and electromyography (EMG) shows no myopathic changes [2]. Yet, in some persons with NS‐PME, nerve conduction studies (NCS) revealed signs of sensory neuronopathy and anterior horn cell involvement [2, 13]. This has not been reported in the other three *GOSR2* genotypes associated with the classical phenotype, but only one person underwent NCS and EMG [18]. Other features that were only reported in NS‐PME include hypothyroidism (*n* = 3), delayed puberty (*n* = 2) and optic atrophy (*n* = 1) [2, 3].

Treatment is mainly aimed at myoclonus and seizure suppression by a combination of 3–5 anti‐seizure medications (ASM), mostly including levetiracetam, clonazepam and valproic acid. Despite multiple ASMs, debilitating myoclonic jerks and seizures persist [2]. Other therapies that have been applied in NS‐PME include ketogenic diet and deep brain stimulation (DBS) [14, 26]. Ketogenic diet was investigated in four persons with NS‐PME and led to improvement of health related quality of life in one perrson, who continued with ketogenic diet [26].

### 3.3 Predominant Congenital Muscular Dystrophy (CMD) Phenotype

Another phenotype of mutations in *GOSR2* is congenital muscular dystrophy (CMD). One person who is compound heterozygous for the known c.430G>T and a novel c.82C>T (p.Gln28Ter) mutation showed a predominant CMD phenotype [21]. She was reported to have axial and peripheral hypotonia, global developmental delay and persistently elevated CK levels (range 459–886 U/L). Muscle biopsy was not performed. Nystagmus was found on ophthalmology exams, however no other neurological involvement such as myoclonus or ataxia was reported. There were no clinical seizures at the last follow‐up at age 18 months, however EEG was suggestive of interictal activity.

### 3.4 Mixed Phenotype: CMD And PMA/PME

Four cases with compound heterozygous mutations in *GOSR2* displayed this mixed phenotype. All were compound heterozygous for the known c.430G>T mutation combined with a novel c.336 + 1G>A (p.?) (*n* = 2), c.2 T>G (p.Met1Arg) (*n* = 1) or c.22dup (p.Thr8fs) (*n* = 1) mutation [4, 17, 19, 23].

The mixed phenotype presents with hypotonia, muscle weakness and developmental delay in the first year of life. CK levels are consistently reported to be elevated between 833 and 5582 U/L. Clinical seizures become evident around the age of 2–3 years and consist of both focal and generalized (myoclonic, atonic, absences) seizures and status epilepticus, which rapidly progress to treatment refractory seizures. EEG shows occipital spikes and wave discharges and intermittent rhythmic delta activity with photosensitivity. EMG was conducted in one case (compound heterozygous for c.430G>T and c.336 + 1G>A) and demonstrated myopathic changes. In two cases, muscle biopsies revealed dystrophic changes and absent or hypoglycosylation of dystroglycan (compound heterozygosity for c.430G>T + c.336 + 1G>A and c.430G>T + c.2T>G). In contrast, one muscle biopsy showed normal alpha‐dystroglycan levels but exhibited non‐dystrophic changes (compound heterozygosity for c.430G>T + c.336 + 1G>A). The person with the compound heterozygous mutation c.430G>T and c.22dup also exhibited myoclonus and scoliosis. Orthopedic complications, myoclonus and ataxia were not described in the other three cases with the mixed phenotype. Cognitive decline was reported in one person who is compound heterozygous for c.430G>T + c.22dup.

### 3.5 Hearing Loss

A recent report described a family where four children—three from one set of parents and one from another—were affected by profound congenital hearing loss. The two sets of parents are each first cousins (consanguineous) but all parents have normal hearing. These children were found to be homozygous for the c.1A>C (p.Met1Leu) mutation [5]. Symmetric sensorineural hearing loss was also reported for a person who is compound heterozygous for *GOSR2* mutations (the novel c.204‐7A>G; p.? and c.319C>T; p.Arg107*), which began at age 5 years and progressed slowly [22]. Accompanying features differ in the group with a hearing loss phenotype. Whereas the four children homozygous for the c.1A>C mutation all had febrile seizures but no other (neurological) involvement, the person with the compound heterozygous c.204‐7A>G + c.319C>T mutation had the mixed PMA‐PME with CMD phenotype alongside hearing loss. She initially presented with frequent falls and developed myoclonus epilepsy at age 7 and ataxia at age 13. Additionally, she presented with areflexia and muscle weakness and showed mild cognitive decline in her fifth decade. Muscle biopsy showed hyperglycosylation of alpha‐dystroglycan. Her symptoms thus involve all GOSR2‐associated phenotypes: mixed PMA‐PME, CMD, and hearing loss.

### 3.6 GOSR2 Associated Cardiovascular Risk Factors

The literature review also revealed that in total, 15 Single Nucleotide Variants (SNV) in *GOSR2* have been associated with a range of cardiovascular and metabolic profiles, primarily leading to increased risk of hypertension, myocardial infarction and anatomical cardiovascular anomalies (see Table S1).

## 4 Current Understanding of GOSR2: A SNARE Protein in the Secretory Pathway

**FIGURE 2:**
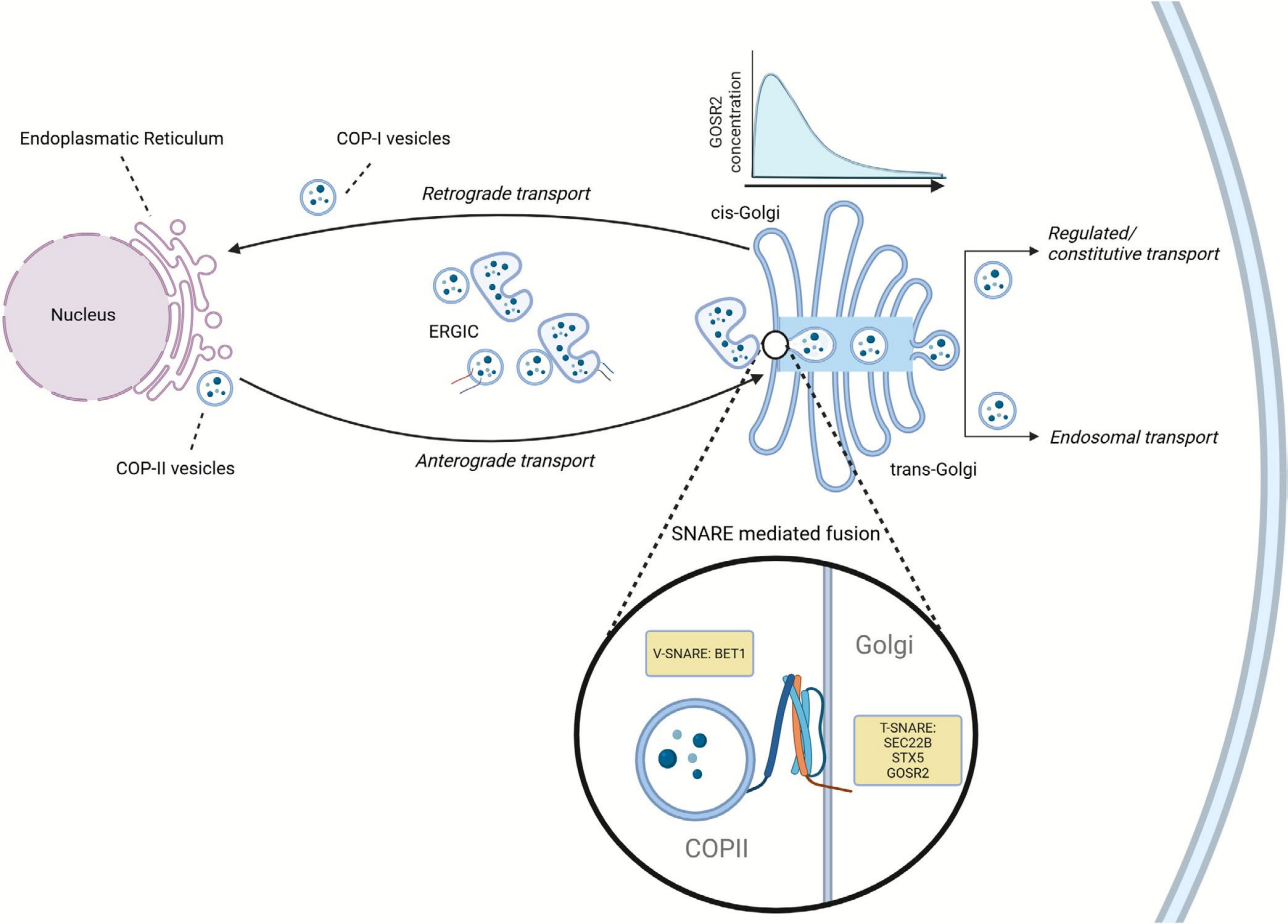
Overview of the secretory pathway with a focus on the SNARE‐mediated fusion by the quaternary complex of sec22b, syntaxin‐5, bet1 and GOSR2. The secretory pathway begins as COP‐II vesicles transport cargo from the endoplasmic reticulum (ER) to the ER‐Golgi intermediate compartment (ERGIC). In the ERGIC, vesicles merge with others before proceeding toward the Golgi apparatus. To fuse with the cis‐Golgi, this process requires a SNARE‐mediated fusion mechanism. Specifically, a quaternary SNARE complex, composed of Sec22b, Syntaxin‐5 (Stx5), Bet1, and GOSR2, facilitates this fusion. Proteins that are not yet ready for Golgi processing are redirected back by COP‐I vesicles through the retrograde transport pathway. SNARE proteins, such as GOSR2, can also enter this recycling pathway as needed. As vesicles progress through the medial and trans‐Golgi regions, they are directed into one of three pathways: regulated secretion, constitutive secretion, or endosomal transport. Notably, GOSR2 levels decrease from the cis‐Golgi toward the trans‐Golgi, suggesting that GOSR2 is primarily active in transport from the ERGIC to the cis‐Golgi site. Created in BioRender by Polet S (https://BioRender.com/uuuxy6v).

Vesicle fusion is crucial in the cellular secretory pathway, ensuring precise delivery of newly synthesized proteins and lipids to their target destinations for proper cellular function. Within the secretory pathway, proteins and lipids are synthesized and modified in the Endoplasmatic Reticulum (ER) and thereafter transported to the cis‐Golgi in COPII‐coated vesicles. During this transport, they pass through the ER‐Golgi intermediate compartment (ERGIC). In the ERGIC, vesicles cluster and are shuttled along with their cargo toward the cis‐Golgi. This entire process is referred to as the anterograde transport route (Figure 2). The fusion of COPII vesicles with the cis‐Golgi membrane, facilitated by a quaternary SNARE complex, is essential for further protein processing [27]. After transiting through the Golgi and arriving at the trans‐Golgi site, proteins and lipids undergo final sorting before being packaged into vesicles. These vesicles are then directed either to the plasma membrane or the cell surface (constitutive transport/regulated transport) or to endosomes [28, 29].

GOSR2 is mainly localized to the cis‐Golgi and has been observed to decrease in concentration as it approaches the trans‐Golgi [30]. This suggests that GOSR2 is primarily involved in facilitating transport of vesicles arriving at the cis‐Golgi, traveling toward the trans‐Golgi site. However, GOSR2 has also been detected in the ERGIC [30, 31]. Furthermore, it has been suggested that GOSR2 is recycled after cis‐Golgi membrane fusion and is re‐used by COP‐I coated vesicles, which are key players of the retrograde transport [31, 32] (Figure 2). Retrograde transport plays a crucial role in recycling proteins essential for fusion, such as SNARE proteins, and returning cis‐Golgi proteins to the ER for quality control [33].

**FIGURE 3:**
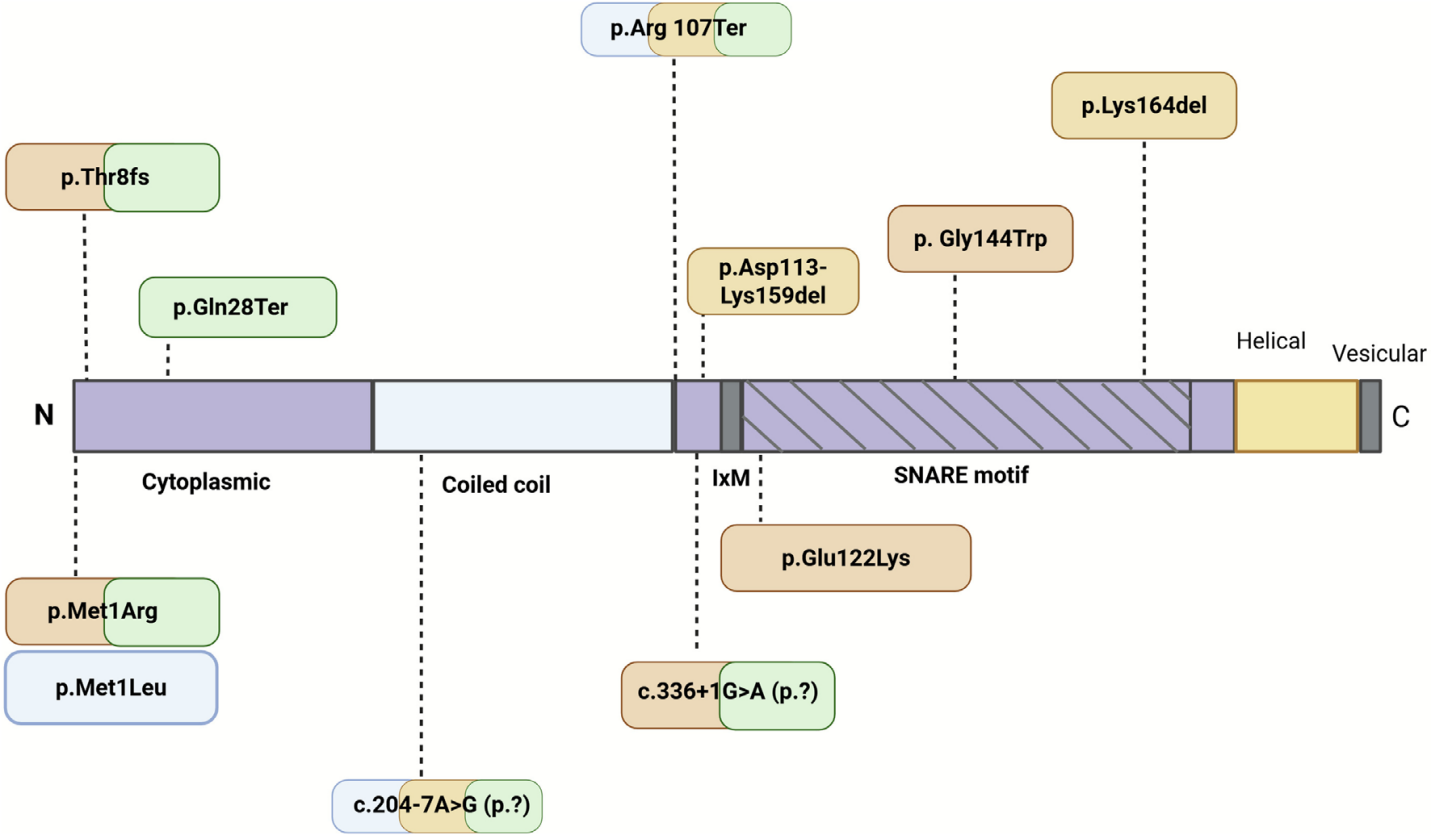
Overview of the domains of the GOSR2 protein, with the reported *GOSR2* mutations. The mutations are linked to the phenotypes by an orange (=PMA/PME phenotype), green (=CMD phenotype) and/or blue (=hearing loss) box. The GOSR2 protein consists of the cytoplasmic domain, the region of the protein that is oriented toward the cytosol, the transmembrane region (helical domain) and the luminal region (the vesicular domain which is oriented toward the Golgi lumen). The coiled coil domain mediates structural stability whereas the IxM motif is critical for the incorporation of GOSR2/membrin and syntaxin‐5 into COPII‐coated vesicles. The SNARE domain is responsible for the assembly of the quaternary SNARE complex of GOSR2 and it's binding partners syntaxin‐5, bet1 and sec22b. Created in BioRender by Polet S (https://BioRender.com/wm0zdsj).

GOSR2 is classified as a target‐SNARE (t‐SNARE) and contains an essential SNARE domain that drives vesicle fusion (Figure 3). It partners with two other t‐SNAREs, Sec22b and syntaxin‐5, to form the t‐SNARE complex on the cis‐Golgi membrane. Fusion between COPII vesicles and the cis‐Golgi membrane is facilitated by the formation of a quaternary complex called a “SNARE pin”, consisting of a t‐SNARE complex (GOSR2, Sec22b, and syntaxin‐5) with Bet1, the vesicle‐SNARE (v‐SNARE) located on COPII vesicles [34, 35]. These “SNARE pins” are formed through interactions between the SNARE domains on each of the SNARE proteins (GOSR2, Sec22b, syntaxin‐5 and Bet1). The SNARE domains contain 15 hydrophobic residues with a central hydrophilic arginine or glutamine. Upon proximity of each other, these SNARE domains “zipper up” through interactions between their layers, enabling membrane fusion (Figure 2) [10, 36].

## 5 Molecular and Cellular Effects of Mutations in *GOSR2*

The most prevalent mutation in *GOSR2* is the homozygous missense mutation c.430G>T and is located within the functional SNARE domain of GOSR2 (Figure 3). Experiments with yeast carrying the orthologous Bos1 p.Gly176Trp showed severe growth defects with minimal growth after 72 h compared to wildtype [15]. In the same study, physical simulations showed that substitution of the small glycine to the larger tryptophan destabilized the hydrophobic core of the SNARE domain which might interfere with the assembly of the SNARE pin and thereby reduce vesicle fusion activity [15]. In the yeast mutant Bos1 p.Gly176Trp, vesicle fusion rates were significantly reduced [37]. However, fusion rates were higher compared to a negative control in which the v‐SNARE Bet1 was omitted. Furthermore, in fibroblasts of persons with NS‐PME, GOSR2 retained the capability to localize to the cis‐Golgi. Together with the findings of Völker et al. who showed impaired, but not absent, growth in *bos1* mutants [15]; this suggests that the homozygous c.430G>T mutation causes a partial loss of function of GOSR2 [37]. The partial loss of function is further demonstrated in section 4 on cellular and animal models.

The persons with different compound heterozygous *GOSR2* mutations showed a variety in disease phenotype. Praschberger et al. reported a case with compound heterozygous mutations, c.430G>T and a novel c.491_493delAGA, which resulted in a milder phenotype compared to NS‐PME [18]. In yeast, the orthologous mutation in Bos1 showed that the c.491_493delAGA mutation led to less severe growth impairment compared to the c.430G>T mutation [15]. The c.491_493delAGA mutation also occurs within the functional SNARE domain; however, simulations indicate that it does not compromise the integrity of the hydrophobic core, unlike the c.430G>T mutation. Instead, it is predicted to disrupt hydrogen bonding between GOSR2 and syntaxin‐5, resulting in a less pronounced effect on SNARE pin assembly and vesicle fusion activity [15].

Stemmerik et al. described a 48‐year‐old person who was compound heterozygous for the c.319C>T and c.204‐7A>G mutations and had with milder disease progression compared to classical NS‐PME [22]. The c.319C>T nonsense mutation, located at the coiled‐coil and cytoplasmic domain boundary (Figure 3), is predicted to cause a loss of function [22]. The c.204‐7A>G mutation likely creates an alternative 3′ splice site, resulting in about 40% abnormal splicing, which was verified by cDNA analysis of muscle‐derived RNA. However, recently the c.204‐7A>G was classified as likely benign in ClinVar. This, or the residual normal splicing, may explain the slower progression to wheelchair dependency compared to NS‐PME. SpliceAI predicts that the c.204‐7A>G mutation induces a new acceptor site (acceptor gain, delta score 0.98) in intron 3 (available on https://spliceailookup.broadinstitute.org/). This could lead to intron inclusion by the activation of a cryptic splice site. However, another splice site mutation reported in *GOSR2* (c.336 + 1G>A) leads to loss of the canonical donor splice site of intron 4 (SpliceAI delta score 0.99), which usually leads to exon skipping. Thus, a similar type of a mutation, in this case a splice site mutation, may lead to varying degrees of disease severity depending on the effects on acceptor or donor sites and subsequent exon skipping or intron inclusion in pre‐mRNA.

Aburayyan et al. studied two mutations occurring in close proximity on the *GOSR2* gene, yet resulting in different clinical phenotypes [5]. The mutation c.1A>C (p.Met1Leu) permits partial translation and is associated with hearing loss but no neurological symptoms. In contrast, the c.2T>G (p.Met1Arg) mutation completely abolishes translation, leading to severe muscular dystrophy and refractory epilepsy. This disparity may be explained by the structural and chemical differences between the substitutions: the side chain of arginine is substantially bulkier and positively charged, whereas both methionine and leucine are non‐polar and hydrophobic, making leucine more chemically similar to methionine [38]. It remains intriguing, however, why there is no hearing loss in the p.Met1Arg mutation considering the large impact on translation of GOSR2.

**FIGURE 4:**
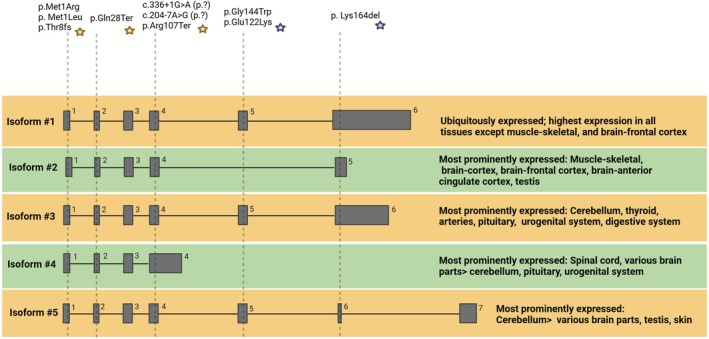
Overview of the five main isoforms of GOSR2 according to GTEx Analysis Release V8 (dbGaP Accession phs000424.v8.p2) with genome build GRCh38. For simplicity, isoforms are labeled #1–5, with the official names of the isoforms as follows: Isoform #1 = ENST00000640051.1; Isoform #2 = ENST00000393456.7; Isoform #3 = ENST00000638838.1; Isoform #4 = ENST00000575949.6; Isoform #5 = ENST00000573224.2. To indicate which mutations affect which exon and isoform, their approximate position has been indicated. Yellow stars indicate mutations primarily associated with a Congenital Muscular Dystrophy (CMD) phenotype, mutations with purple star indicate phenotypes primarily associated with the classical Progressive Myoclonus Epilepsy (PME)‐Progressive Myoclonus Ataxia (PMA) phenotype. Created in BioRender by polet S (https://BioRender.com/m5pekxa).

The location of mutations within the GOSR2 gene differentially impacts related isoforms, which further contributes to disease variability. GOSR2 has multiple isoforms, ranging from 2 to 10 exons, with isoform 2 being expressed predominantly in skeletal muscle. Mutations linked to PME without CMD typically occur in exons 5 or 6 and therefore don't affect isoform 2 (Figure 4) [3, 16]. In contrast, mutations associated with CMD often disrupt exons 1 or 4 and affect all five major isoforms [4, 17, 19, 21, 22, 23]. When considering the location of mutations on the GOSR2 protein domains (Figure 3), it is notable that the PME associated mutations are located on the SNARE motif, while the CMD associated mutations are mainly located in the coiled coil and cytoplasmic domain.

Altogether, the type of mutation (e.g., splice site) or location (e.g., start codon) does not always predict phenotypic variability, as illustrated by the reports of Aburayyan et al. and Stemmerik et al. [5, 22]. Instead, disease variability may be explained by differences in impairment of GOSR2 function, which is partially linked to the degree of alteration in protein structure and SNARE binding capacity. Notably, *GOSR2* mutations linked to CMD impact all major isoforms, whereas mutations associated with PME without CMD do not affect the muscle‐specific isoform of *GOSR2*.

Notably, a starting‐site specific mutation in one of the SNARE partners of GOSR2, syntaxin‐5, has been shown to lead to the loss of one specific isoform [39]. This isoform turned out to be the most important isoform for interaction with Bet1 and was differently expressed among various tissues [39]. Similarly, mutations in *Bet1*, the v‐SNARE partner of GOSR2, have been described [38]. Both mutations in *Bet1* and *syntaxin‐5* are associated with early fatal multisystem involvement and defects in glycosylation.

## 6 Pathophysiological Insights Derived From Cellular and Animal Studies With Altered *GOSR2* Function/Levels

*Drosophila melanogaster* (fruitfly) is a highly versatile model organism, frequently used to study genetic disorders due to their well‐characterized genome, short life cycle, and ease of manipulation in research. The fruitfly has an ortholog of *GOSR2*, referred to as Membrin. Several Membrin alterations in fruitfly models lead to disturbances in development toward the adult stage. The life cycle of a fruitfly starts with the embryonic development followed by hatching from the egg. Then, the larval period starts with three distinct stages (L1, L2, L3) and subsequently a pupal period. In the pupal phase, the larva transforms into a pupa with a hard outer case, during which adult fly features such as wings, legs and eyes develop. Lastly, adult flies emerge from their pupal cases, a process referred to as eclosion. Praschberger et al. have shown that a full knockout of Membrin in fruitflies leads to lethality prior to the L2 larval stage [37]. Subsequently, Praschberger et al. created mutant transgenic *GOSR2* fly lines, displaying G144W (referring to c.430G>T) and K164del (referring to c.491_493delAGA) mutations. The mutant fly lines survived significantly longer than the flies without Membrin. However, the mutant *Membrin* flies often died in the pupal cases, and in case they survived to adulthood, they exhibited severe locomotor defects and early lethality. Overexpression of the mutant *Membrin* in neurons led to the same eclosion defects as seen in the mutant *Membrin* lines. Mutant *Membrin* fly lines further showed reduced dendritic growth and synaptic trafficking, presynaptic and axonal morphological changes and reduced spontaneous neurotransmission [37].

Knockdown (as opposed to knock‐out) of Membrin in all cells of the fruitfly led to male lethality and decreased survival until adulthood in female fruitflies. Additionally, female fruitflies displayed heat‐induced seizures [24]. Knockdown of Membrin specifically in glial cells led to normal numbers of offspring in males and females, and these flies also displayed heat‐induced seizures, progressing with aging. Interestingly, knockdown of Membrin in neuronal cells did not lead to a heat‐induced seizure phenotype, suggesting a pivotal role of glial cells in the pathophysiology of hyperexcitability in GOSR2‐related disorders [24]. It is still unclear why the mutant *Membrin* fruitfly points toward neuronal abnormalities (e.g., axonal morphological changes), while the knockdown phenotype was only prominent in glial cells and not in neurons [24, 37]. Furthermore, the GOSR2‐glial knockdown fruitfly model showed significant seizure reduction with anti‐seizure medications working on the GABA‐A receptor (barbital, clonazepam and ganaxolone) [40]. This suggests a specific role of post‐excitatory GABA‐ergic inhibition in the occurrence of hyperexcitability in GOSR2‐related disorders.

Similar to flies, the International Mouse Phenotyping Consortium demonstrated that homozygous knockout of GOSR2 in mice results in embryonic lethality (Home|IMPC|International Mouse Phenotyping Consortium [https://www.mousephenotype.org/]) [41]. The consortium linked heterozygous knockout of GOSR2 to gait abnormalities, aligning with the predominantly neurological phenotype observed in humans. Heterozygous knockout of GOSR2 was also associated with modulation of cardiac conduction in mice, based on increased conduction velocity and both a shortened action potential duration and maximal upstroke velocity. These cardiac conduction abnormalities are thought to be the consequence of the modulating effect of Membrin on a cardiac sodium channel (NaV 1.5) [42].

## 7 Discussion

The phenotypic and genotypic spectrum of GOSR2‐associated conditions has expanded in the past 10 years. In contrast to what was previously described, we here show that mutations in *GOSR2* do not solely lead to movement disorders and epilepsy, but are associated with a wide spectrum of neurological features and multisystem involvement, which fits with the ubiquitous function of GOSR2. The three main phenotypes associated with *GOSR2* mutations are: (1) Progressive Myoclonus Epilepsy/Ataxia (PMA/PME), (2) Congenital Muscular Dystrophy (CMD) and (3) Hearing loss. Mutations in *GOSR2* are presumed to lead to a partial loss‐of‐function, with GOSR2 still contributing to SNARE complex formation at its main “working site”: the cis‐Golgi. This corresponds with the finding in animal models, which have shown that some GOSR2 activity is needed for viability. Disease variability is associated with the structural effects on GOSR2 and GOSR2 incorporation into the four‐bundle SNARE complex. Even mutations similar in type (e.g., splice site) or location can have distinct impacts on this functionality, leading to different disease outcomes.

The mutations affecting exon 1 and 4 are mainly associated with CMD, while PMA/PME associated mutations mainly affect exon 5, thereby sparing the GOSR2 isoform which is highly expressed in the muscular‐skeletal system. Future studies should further explore the role of the various GOSR2 isoforms in more detail. Recently, a starting site‐specific variant in syntaxin‐5 (one of the SNARE partners of GOSR2) was shown to lead to the loss of specifically the short isoform of syntaxin‐5. This isoform was found to have a dominant role in retrograde (Golgi‐ER) and intra‐Golgi trafficking and engages differently in the formation of SNARE complexes compared to the long isoform. The loss of the short isoform of syntaxin‐5 can be partially compensated by the long isoform, with sufficient anterograde trafficking until the Golgi, but impaired intra‐ and post Golgi trafficking [39]. In addition, ER and Golgi morphology was altered due to the loss of the short isoform [39]. It would be of particular interest to identify whether there are dominant or specific isoforms of GOSR2 for anterograde or retrograde SNARE trafficking and whether these are associated with altered Golgi and ER morphology e.g., through patient‐fibroblast studies. Additionally, the expression of GOSR2 isoforms in specific tissues deserves further evaluation. This may provide insight into why the c.2T>G (p.Met1Arg) mutation does not result in hearing loss, despite significant neurological involvement. Perhaps, there is a specific/dominant isoform of *GOSR2* for hair cells, which is not affected by the c.2T>G (p.Met1Arg) mutation but significantly affects the nervous system. Collectively, identifying the impact of *GOSR2* mutations on the different isoforms and their cellular functions will improve understanding of genotype–phenotype associations in GOSR2‐associated conditions and may contribute to advancing targeted therapies.

Glycosylation defects (mainly hypoglycosylation) have been reported in several of the compound heterozygous *GOSR2* variants. Furthermore, glycosylation defects were also reported in mutations of the binding partners of GOSR2 (Bet1 and syntaxin‐5) [39, 43]. However, glycosylation defects have not yet been associated with the most common mutation in *GOSR2* (c.430G>T; p.Gly144Trp). Studying fibroblasts and muscle cells from persons with NS‐PME to determine the presence of glycosylation defects could provide crucial insights into the disease mechanisms and potentially redefine GOSR2‐associated disorders as part of the Congenital Disorders of Glycosylation (CDG) spectrum.

Interestingly, in the classical PMA‐PME phenotype, not only fever but also external heat (e.g., bathing) has been reported to trigger myoclonus and seizures. A fruitfly model with a temperature sensitive SNARE mutant (SNAP‐25 mutant) was shown to have more competent SNARE complex fusion at lower temperatures, and SNARE instability at higher temperatures [44]. Perhaps, the same accounts for the *GOSR2* mutants, with SNARE complex assembly being less effective at higher temperatures. Interestingly, intercurrent illness as a trigger is particularly relevant given the central role of glial cells in the NS‐PME fruitfly model. In this model, the ortholog of Membrin was downregulated in both neurons and glial cells. Notably, downregulation in neurons alone did not produce a phenotype. However, when Membrin was specifically downregulated in glial cells, flies exhibited a progressive, heat‐induced seizure phenotype. This strongly suggests that Membrin/GOSR2 dysfunction primarily impairs glial cell function. In view of the fever/intercurrent illness as a trigger of symptom exacerbation, it would be interesting to investigate which glial cells are involved in the pathophysiology of NS‐PME; especially whether microglia plays a central role in NS‐PME. Microglia are the primary glial cells involved during an infection and, when activated due to an infection, release cytokines which can result in neurotransmitter disturbances and neuronal hyperexcitability [45, 46]. Future studies should investigate which glial cells are involved in the pathophysiology of NS‐PME.

Current treatment modalities of *GOSR2‐*associated conditions offer limited and unsatisfactory symptom control. Ganaxolone, a new GABA‐A targeting antiseizure drug, warrants evaluation for seizure and myoclonus suppression. The advancement of genetic techniques including genome editing could open new therapeutic possibilities for rare monogenic disorders like *GOSR2‐*associated conditions. To advance gene therapy for GOSR2‐related disorders, a deeper understanding is required of the specific tissues and cell types involved—such as the particular glial cells affected—as well as the extent of functional loss of SNARE complexes in *GOSR2* mutants. Additionally, determining the therapeutic threshold, or the level of *GOSR2* restoration necessary for meaningful SNARE function restoration and clinical benefit is essential for developing interventions with expected clinical relevance. In this respect, patient‐derived cell models like induced pluripotent stem cells (iPSCs), neural progenitor cells and/or organoids may provide powerful disease models, enabling detailed investigations of pathophysiological mechanisms, high‐throughput drug screening, and the development of gene‐editing strategies in a patient‐derived and organ‐specific setting. This might also help us understand why the neurological system is predominantly affected by mutations in *GOSR2*.

In conclusion, GOSR2 is a ubiquitously expressed protein with a critical role in the vesicle trafficking pathway. This is illustrated by the wide spectrum of neurological and non‐neurological manifestations and the severe disease progression associated with GOSR2‐related disorders. While mutations in *GOSR2* were initially linked primarily to PMA/PME, they should also be considered in persons with hearing loss, congenital muscular dystrophy (CMD), or a combination of these phenotypes. The findings in our study suggest a genotype–phenotype correlation, particularly in relation to SNARE complex disruption and isoform‐specific effects. However, important questions remain, including the reason for a predominant neurological phenotype, the role of specific isoforms in disease pathophysiology, and the broader impact of GOSR2 dysfunction on ER‐Golgi trafficking. Use of patient‐specific disease modeling may advance targeted therapeutic strategies for GOSR2‐associated disorders.

## Author Contributions

**Sjoukje S. Polet:** conceptualization, data curation, formal analysis, investigation, methodology, project administration, visualization, writing – original draft. **Elisabeth Z. Siegal:** conceptualization, data curation, validation, formal analysis, visualization, writing – review and editing. **Sabine A. Fuchs:** validation, visualization, supervision, writing – reviewing and editing. **Marina A. J. Tijssen:** validation, methodology, visualization, supervision, writing – reviewing and editing. **Tom J. de Koning:** conceptualization, visualization, validation, data curation, planning, investigation, methodology, supervision, writing – review and editing.

## Ethics Statement

No ethics approval was obtained since the work consisted of a narrative literature search, for which no ethical approval is necessary in the institutions/countries participating in the study.

## Conflicts of Interest

Marina A. J. Tijssen reports grants from the Netherlands Organisation for Health Research and Development Domain: NWO‐TTW (2022–9), ZonMW Topsubsidie (91218013) and ZonMW Program Translational Research (40‐44600‐98‐323). She also received two European Funds for Regional Development from the European Union (01492947 & DIMATIO (EFRO‐0059)) and a European Joint Programme on Rare Diseases (EJP RD) Networking Support Scheme. A grant from Health Holland and the PPP allowance program (PPP‐2023‐00). Furthermore, grants from the province of Friesland, the Stichting Wetenschapsfonds Dystonie, Epilepsie NL (WAR 25‐07) and unrestricted grants from Ipsen, Actelion and Merz. Sabine A. Fuchs received a ZonMW VICI grant, an ERC Starting Grant, an ERC Proof of Concept Grant, and a KNAW Ammodo Science Award, all focused on prime editing. She is also a recipient of two TKI‐HH grants related to gene editing, each co‐funded by a company: Takeda and TriLink. Tom J. de Koning received an unrestricted grant from PTC Pharmaceuticals and a grant from the Dutch Brain Foundation. He is a medical advisor of the Metabolic Power Foundation (Stichting Stofwisselkracht, non‐profit), the North Sea Disease Foundation (Stichting Noordzeeziekte, nonprofit), Janivo Foundation (Stichting Janivo, nonprofit), and Ancora Health bv (profit). He has shares in Ancora Health bv. Elisabeth Z. Siegal and Sjoukje S. Polet have nothing to declare.

## Supporting information


**FIGURE S1:** jimd70115‐sup‐0001‐FigureS1.pdf.

**FIGURE S2:** jimd70115‐sup‐0002‐FigureS2.pdf.

**FIGURE S3:** jimd70115‐sup‐0003‐FigureS3.pdf.

**FIGURE S4:** jimd70115‐sup‐0004‐FigureS4.pdf.

**TABLE S1:** Overview of single nucleotide variants (SNV) in *GOSR2* associated with cardiovascular risks and anatomical variations.

## Data Availability

Data sharing not applicable to this article as no datasets were generated or analysed during the current study.
