# Supplementary material for: The Genotypic and Phenotypic Spectrum of GOSR2 Mutations: Clinical and Pathophysiological Insights
Source: J Inherit Metab Dis. 2025 Nov 20;48(6):e70115. doi: 10.1002/jimd.70115 (PMC12631098; doi:10.1002/jimd.70115)
Supplement: Supplementary file 5 — TABLE S1: Overview of single nucleotide variants (SNV) in GOSR2 associated with cardiovascular risks and anatomical variations. [file JIMD-48-0-s004.docx]

| SNV | |  | Association | ^Reference^ |
| --- | --- | --- | --- | --- |
| SNVs  rs17677363, rs11874, and rs76774446 | |  | Associated with Increased risk of anomalies of thoracic arteries and veins (ATAV) | Lahm et al, 2021 [1] |
| SNV  rs17608766 | |  | Associated with systolic and diastolic blood pressure regulation | 2011, consortium [2] |
| SNV  rs197922 | |  | Associated with Hypertension in whites/Caucasians? | Meyer et al, 2009 [3] |
| T-G-G haplotype (rs197932-rs3785889-rs197922 | |  | Associated with lower risk for myocardial infarction in Japanese men | Pan et al, 2013 [4] |
| G-T haplotype established by rs3785889-rs16941382 | |  | May be genetic risk factor for CAD in Chinese Han population | Pan et al, 2017 [5] |
| SNV  rs1052586 | |  | Associated with susceptibility to myocardial infarction | Yamada et al, 2018 [6] |
| SNV  rs197922 |  | | associated with systolic blood pressure, mean arterial pressure and hypertension in East Asian population. | Xing-Bo Mo et al, 2019 [7] |
| SNV  rs17608766 | |  | Associated with aortic root diameter association | Wild et al, 2017 [8] |
| SNV  rs17608766 | |  | Associated with pulse pressure regulation | Simino et al, 2014 [9] |
|  | |  | GOSR2 associated as cataract susceptibility gene | Choquet et al, 2024. [10] |
| SNV  rs17608766 | |  | Associated with decreased risk of T2D in CAD | Al Hageh et al, 2024 [11] |
| T–A–G haplotype (rs197932–rs3785889–rs197922) | |  | Associated with hypertension in Japanese men | Pan et al, 2013 [12] |
| SNV rs6504673  SNV rs533030436 | |  | Associated with variations in mitral valve annular diameter in systole  Associated with variations in mitral valve annular diameter in diastole | Yu et al, 2022 [13] |

**Supplementary table 1.** Overview of single nucleotide variants (SNV) in *GOSR2* associated with cardiovascular risks and anatomical variations.

**References supplementary material**

[1] Lahm H, Jia M, Dreßen M, Wirth F, Puluca N, Gilsbach R, Keavney BD, Cleuziou J, Beck N, Bondareva O, Dzilic E, Burri M, König KC, Ziegelmüller JA, Abou-Ajram C, Neb I, Zhang Z, Doppler SA, Mastantuono E, Lichtner P, Eckstein G, Hörer J, Ewert P, Priest JR, Hein L, Lange R, Meitinger T, Cordell HJ, Müller-Myhsok B, Krane M. Congenital heart disease risk loci identified by genome-wide association study in European patients. J Clin Invest. 2021 Jan 19;131(2):e141837. doi: 10.1172/JCI141837.

[2] International Consortium for Blood Pressure Genome-Wide Association Studies. Genetic variants in novel pathways influence blood pressure and cardiovascular disease risk. Nature. 2011 Sep 11;478(7367):103-9. doi: 10.1038/nature10405.

[3] Meyer TE, Shiffman D, Morrison AC, Rowland CM, Louie JZ, Bare LA, Ross DA, Arellano AR, Chasman DI, Ridker PM, Pankow JS, Coresh J, Malloy MJ, Kane JP, Ellis SG, Devlin JJ, Boerwinkle E. GOSR2 Lys67Arg is associated with hypertension in whites. Am J Hypertens. 2009 Feb;22(2):163-8. doi: 10.1038/ajh.2008.336.

[4] Pan S, Nakayama T, Sato N, Izumi Y, Soma M, Aoi N, Ma Y, Hinohara S, Doba N. A haplotype of the GOSR2 gene is associated with myocardial infarction in Japanese men. Genet Test Mol Biomarkers. 2013 Jun;17(6):481-8. doi: 10.1089/gtmb.2012.0379.

[5] Pan S, Guan GC, Lv Y, Liu ZW, Liu FQ, Zhang Y, Zhu SM, Zhang RH, Zhao N, Shi S, Nakayama T, Wang JK. G-T haplotype established by rs3785889-rs16941382 in *GOSR2* gene is associated with coronary artery disease in Chinese Han population. Oncotarget. 2017 Jul 17;8(47):82165-82173. doi: 10.18632/oncotarget.19280.

[6] Yamada Y, Kato K, Oguri M, Horibe H, Fujimaki T, Yasukochi Y, Takeuchi I, Sakuma J. Identification of 13 novel susceptibility loci for early-onset myocardial infarction, hypertension, or chronic kidney disease. Int J Mol Med. 2018 Nov;42(5):2415-2436. doi: 10.3892/ijmm.2018.3852.

[7] Mo XB, Lei SF, Zhang YH, Zhang H. Examination of the associations between m^6^A-associated single-nucleotide polymorphisms and blood pressure. Hypertens Res. 2019 Oct;42(10):1582-1589. doi: 10.1038/s41440-019-0277-8.

[8] Wild PS, Felix JF, Schillert A, Teumer A, Chen MH, Leening MJG, Völker U, Großmann V, Brody JA, Irvin MR, Shah SJ, Pramana S, Lieb W, Schmidt R, Stanton AV, Malzahn D, Smith AV, Sundström J, Minelli C, Ruggiero D, Lyytikäinen LP, Tiller D, Smith JG, Monnereau C, Di Tullio MR, Musani SK, Morrison AC, Pers TH, Morley M, Kleber ME, Aragam J, Benjamin EJ, Bis JC, Bisping E, Broeckel U, Cheng S, Deckers JW, Del Greco M F, Edelmann F, Fornage M, Franke L, Friedrich N, Harris TB, Hofer E, Hofman A, Huang J, Hughes AD, Kähönen M, Investigators K, Kruppa J, Lackner KJ, Lannfelt L, Laskowski R, Launer LJ, Leosdottir M, Lin H, Lindgren CM, Loley C, MacRae CA, Mascalzoni D, Mayet J, Medenwald D, Morris AP, Müller C, Müller-Nurasyid M, Nappo S, Nilsson PM, Nuding S, Nutile T, Peters A, Pfeufer A, Pietzner D, Pramstaller PP, Raitakari OT, Rice KM, Rivadeneira F, Rotter JI, Ruohonen ST, Sacco RL, Samdarshi TE, Schmidt H, Sharp ASP, Shields DC, Sorice R, Sotoodehnia N, Stricker BH, Surendran P, Thom S, Töglhofer AM, Uitterlinden AG, Wachter R, Völzke H, Ziegler A, Münzel T, März W, Cappola TP, Hirschhorn JN, Mitchell GF, Smith NL, Fox ER, Dueker ND, Jaddoe VWV, Melander O, Russ M, Lehtimäki T, Ciullo M, Hicks AA, Lind L, Gudnason V, Pieske B, Barron AJ, Zweiker R, Schunkert H, Ingelsson E, Liu K, Arnett DK, Psaty BM, Blankenberg S, Larson MG, Felix SB, Franco OH, Zeller T, Vasan RS, Dörr M. Large-scale genome-wide analysis identifies genetic variants associated with cardiac structure and function. J Clin Invest. 2017 May 1;127(5):1798-1812. doi: 10.1172/JCI84840.

[9] Simino J, Shi G, Bis JC, Chasman DI, Ehret GB, Gu X, Guo X, Hwang SJ, Sijbrands E, Smith AV, Verwoert GC, Bragg-Gresham JL, Cadby G, Chen P, Cheng CY, Corre T, de Boer RA, Goel A, Johnson T, Khor CC; LifeLines Cohort Study; Lluís-Ganella C, Luan J, Lyytikäinen LP, Nolte IM, Sim X, Sõber S, van der Most PJ, Verweij N, Zhao JH, Amin N, Boerwinkle E, Bouchard C, Dehghan A, Eiriksdottir G, Elosua R, Franco OH, Gieger C, Harris TB, Hercberg S, Hofman A, James AL, Johnson AD, Kähönen M, Khaw KT, Kutalik Z, Larson MG, Launer LJ, Li G, Liu J, Liu K, Morrison AC, Navis G, Ong RT, Papanicolau GJ, Penninx BW, Psaty BM, Raffel LJ, Raitakari OT, Rice K, Rivadeneira F, Rose LM, Sanna S, Scott RA, Siscovick DS, Stolk RP, Uitterlinden AG, Vaidya D, van der Klauw MM, Vasan RS, Vithana EN, Völker U, Völzke H, Watkins H, Young TL, Aung T, Bochud M, Farrall M, Hartman CA, Laan M, Lakatta EG, Lehtimäki T, Loos RJ, Lucas G, Meneton P, Palmer LJ, Rettig R, Snieder H, Tai ES, Teo YY, van der Harst P, Wareham NJ, Wijmenga C, Wong TY, Fornage M, Gudnason V, Levy D, Palmas W, Ridker PM, Rotter JI, van Duijn CM, Witteman JC, Chakravarti A, Rao DC. Gene-age interactions in blood pressure regulation: a large-scale investigation with the CHARGE, Global BPgen, and ICBP Consortia. Am J Hum Genet. 2014 Jul 3;95(1):24-38. doi: 10.1016/j.ajhg.2014.05.010.

[10] Choquet H, Duot M, Herrera VA, Shrestha SK, Meyers TJ, Hoffmann TJ, Sangani PK, Lachke SA. Multi-tissue transcriptome-wide association study identifies novel candidate susceptibility genes for cataract. Front Ophthalmol (Lausanne). 2024 Apr 16;4:1362350. doi: 10.3389/fopht.2024.1362350.

[11] Al Hageh C, O'Sullivan S, Platt DE, Henschel A, Chacar S, Gauguier D, Abchee A, Alefishat E, Nader M, Zalloua PA. Coronary artery disease patients with rs7904519 (TCF7L2) are at a persistent risk of type 2 diabetes. Diabetes Res Clin Pract. 2024 Jan;207:111052. doi: 10.1016/j.diabres.2023.111052.

[12] Pan S, Nakayama T, Sato N, Izumi Y, Soma M, Aoi N, Ma Y. A haplotype of the GOSR2 gene is associated with essential hypertension in Japanese men. Clin Biochem. 2013 Jun;46(9):760-5. doi: 10.1016/j.clinbiochem.2012.12.021.

[13] Yu M, Tcheandjieu C, Georges A, Xiao K, Tejeda H, Dina C, Le Tourneau T, Fiterau M, Judy R, Tsao NL, Amgalan D, Munger CJ, Engreitz JM, Damrauer SM, Bouatia-Naji N, Priest JR. Computational estimates of annular diameter reveal genetic determinants of mitral valve function and disease. JCI Insight. 2022 Feb 8;7(3):e146580. doi: 10.1172/jci.insight.146580.
